# A prospective association between sensory impairment and cognitive performance among older community‐dwelling adults: The role of depressive symptoms

**DOI:** 10.1002/alz.092490

**Published:** 2025-01-09

**Authors:** Rabia Khalaila, Lauren A Grebe, Isabel Elaine Allen

**Affiliations:** ^1^ Zefat Academic College, Zefat, CA Israel; ^2^ University of California, San Francisco / Global Brain Health Institute, San Francisco, CA USA; ^3^ St. John’s University, Queens, NY USA; ^4^ Department of Epidemiology and Biostatistics, University of California, San Francisco, San Francisco, CA USA; ^5^ Global Brain Health Institute, University of California San Francisco, San Francisco, CA USA; ^6^ Global Brain Health Institute, San Francisco, CA USA; ^7^ Memory and Aging Center, UCSF Weill Institute for Neurosciences, University of California, San Francisco, San Francisco, CA USA; ^8^ University of California San Francisco, San Francisco, CA USA

## Abstract

**Background:**

There is a link between visual and hearing impairments and poor cognition in older people, although it is not clear how depressive symptoms contribute to this association. Specifically, this study examines how sensory impairment (vision and hearing) affects cognitive performance and how depression mediates that effect.

**Method:**

We examined whether vision and hearing impairment affects cognitive performance and whether depression mediates that effect. Utilizing data from the Survey of Health, Ageing and Retirement in Europe which assessed 32,325 participants in 2011 (baseline, Time 1) and 2015 (follow up, Time 2), we analyzed sociodemographic and health factors, self‐reported vision and hearing impairment at baseline, depression, and cognitive performance at the 4‐year follow‐up. A multiple mediator model was tested by bootstrapping with resampling strategies.

**Result:**

At baseline, 22.9% had impaired vision only, 10.2% impaired hearing only, and 10.4% impairment of both. We found significant negative association between vision impairment (b = ‐0.023, p = 0.001) and dual sensory impairment (b = ‐0.083, p = 0.001) and cognitive performance; both were also associated with depression, which in turn was linked with poor cognition.

**Conclusion:**

Visual impairment or dual sensory impairment among older adults are associated with poor cognitive function directly and indirectly by increasing depression symptoms.

